# Influence of Psychological Factors on Participation and Life Satisfaction in the Context of Travel and Tourism after Spinal Cord Injury

**DOI:** 10.3390/ijerph20010516

**Published:** 2022-12-28

**Authors:** Chenggang Hua, Shu Cole

**Affiliations:** Department of Health & Wellness Design, Indiana University School of Public Health-Bloomington, Bloomington, IN 47405, USA

**Keywords:** travel, tourism, life satisfaction, self-determination theory, psychological needs satisfaction, spinal cord injury

## Abstract

Spinal cord injury (SCI) can significantly compromise people’s participation in travel and tourism activities, which is considered an important and meaningful way to engage in one’s chosen lifestyle and wellness pursuits. Yet, travel often presents challenges for people with spinal cord injury (PwSCI), as it requires overcoming a wide range of potential psycho-physical challenges or barriers during trips. There is a lack of theory-based research that can help us understand and address the psychological factors and processes influencing participation and life satisfaction following SCI. Drawing on self-determination theory (SDT), this study examines the effects of psychological needs satisfaction on participation in the travel setting, and their subsequent impact on perceived life satisfaction. This study uses a mixed-methods approach with 39 in-depth telephone interviews conducted that focus on developing needs satisfaction measures for PwSCI in the travel setting, and an online survey among 258 PwSCI examining the relations between needs satisfaction and outcome variables. This study finds that the psychological needs satisfaction of autonomy and relatedness significantly contribute to self-determined participation in travel and tourism activities for PwSCI. This self-determined participation outcome thus represents an individual’s improved ability to exert choice and control, which exhibits their level of regained mobility and further improves their life satisfaction.

## 1. Introduction

Spinal cord injury (SCI) often results in impaired mobility and secondary health conditions (i.e., pain, spasticity, bladder and bowel problems, and pressure ulcers) that restrict the way a person takes part in family, work, and community activities and overall society at large [1]. Research has widely documented the negative effects of a lowered level of participation in various life activities of people after SCI [2], including travel [3]. Travel and tourism have long been considered desirable activities for people with disabilities because they can bring participants pleasure and stimulate their sense of discovery and conviviality [4]. Through travel and tourism activities, participants can fulfill their needs and expectations in daily living [5] by extending their social networks, thus enhancing personal development and full inclusion in society [6]. For people with SCI (PwSCI), the ability to travel is often necessary to return as much as possible to the lifestyle they had before injury [7]. Voluntary travel for tourism represents the ability of this population to exert choice and control in participation, which can help replicate their pre-injury lifestyle. Despite these known benefits, however, some PwSCI forgo travel opportunities due to the multiple challenges they face or anticipate in relation to travel. While much research has focused on the inaccessibility of travel services and facilities for PwSCI, recent research revealed that psychological inhibitors and perceived lack of psychological needs satisfaction play a key role in preventing them from participating in travel and tourism [8]. In this case, neither improving the objective travel settings nor emphasizing the positive outcomes of tourism will necessarily restore travel motivation. Thus, examining the self-determined psychological factors and processes behind the behavior of PwSCI could be effective when fostering their travel participation.

Research has suggested that participation in travel and tourism may contribute to people’s life satisfaction and overall wellbeing [2,5,6] and improve the quality of life of PwSCI. However, there is lack of empirical evidence on the effects of tourism participation on the life satisfaction of PwSCI. To advance our knowledge of how such participation impacts the lives of this population, this study aims to examine the psychological processes related to travel and tourism participation, and how its various antecedents and consequences interact. Guided by self-determination theory (SDT), this study tests hypotheses on the inter-relations of psychological needs satisfaction of autonomy, competence, and relatedness, and their positive consequences, including increased travel and tourism participation and enhanced life satisfaction. Study results can illuminate the psychological factors behind the travel and tourism participation of PwSCI. Specifically, they may reveal aspects of participation that are unique to the population, which can extend self-determination theory (SDT) to this setting. Findings should also offer insights for health professionals as well as travel and tourism service personnel on designing and establishing finely tuned travel and tourism programs based on informed research that enhance the community and social participation experiences of PwSCI.

To give a brief overview of this paper, in Section 2, we carefully review the travel and tourism literature on the travel participation of PwSCI, discuss applications of SDT to PwSCI in the travel context, as well as travel participation’s impacts on the life satisfaction of PwSCI. Based on the literature review, a hypothesized self-determination model of participation in travel and tourism activities for PwSCI is further proposed. In Section 3, we introduce the study’s mixed-method approach, the interview protocols, the scale development process, and quantitative data collection procedures. The results of both qualitative and quantitative analysis are explicitly demonstrated in Section 4. Finally, in Section 5 and Section 6, the study further discusses the results, summary of findings, theoretical and practical implications, and suggestions for future research.

## 2. Literature Review

### 2.1. Travel and Tourism Participation of PwSCI

In this study, travel refers to the activity of travelers (e.g., visitors) moving between different geographic locations for any purpose and duration, with tourism as a subset of travel [9]. Participation, defined as “involvement in life situations” [10], is considered by the International Classification of Functioning, Disability, and Health as a primary rehabilitation outcome for PwSCI. Playing a vital role in this population’s overall health and life experience, the level of participation affects their ability to be an active and contributing member of society after rehabilitation [11]. Research has shown that the ability to travel enhances participation for PwSCI beyond the basic functioning skills to expanded recreational options and other endeavors after injury [12]. Like people without disabilities, PwSCI often need or wish to travel for business, education, health, or leisure, where improving life beyond rehabilitation and re-integrating to the lifestyle experienced before their injury is a major goal [13]. Enhancing this group’s self-determination in travel-related activities is therefore critical for the active pursuit of productive lives as full citizens of their communities and the larger society.

In examining the antecedents to participation in travel and tourism, extant research has largely focused on studying contextual factors that constrain one’s motivation, i.e., [14,15], but intrinsic factors such as self-doubt or fear of failure are often ignored. A major obstacle to participation for people with disabilities is their common perception of travel failures as being inevitable [15]. While studies have analyzed the inaccessible service and environmental barriers affecting the travel decisions of people with disability [16,17], constraint theory further suggests that unless their psychological inhibitors (i.e., hesitation or fear of failures) are resolved [18], they will not be in the position to experience objective environmental inhibitors (i.e., architectural barriers or lack of adapted transportation). In such cases, improving the objective physical travel settings will not necessarily trigger their travel motivation [19]. As psychological inhibitors and lack of psychological needs satisfaction play a key role in preventing them from participating [8], travel among PwSCI could be effectively facilitated by fostering self-determined factors. 

### 2.2. Self-Determination Theory Applications in Travel Participation of PwSCI

The well-established self-determination theory (SDT) has been used in broad research contexts, where its theoretical framework for studies on participation and rehabilitation of PwSCI is increasing in the literature [20]. SDT postulates that it is through interaction with nurturing and supportive factors in our social environment that the three psychological needs of autonomy, competence, and relatedness are fostered and enhanced, which in turn facilitate or hinder motivations and related behaviors in specific social and environmental contexts. Autonomy, or the need to be self-governing and independent, can be achieved when people’s behavior is internally and independently derived, thus satisfying the need for self-initiation; this includes engagement in behaviors that reflect their interests or values. Competence is associated with self-development, as in individuals’ need to feel capable and effective, and responds to the desire to be efficient and influential. Relatedness means to feel connected, accepted, cared for, or bonded with others, and to care for them in return [5,21,22,23]. In the context of this study, the need for relatedness can be satisfied when people feel connected to others during their tourism experiences. 

SDT holds that it is human nature to be intrinsically motivated to partake in behaviors involving challenges, spontaneous interests, exploration, and learning, all of which can be engaged in travel and tourism. SDT refers to autonomy motivation as a core concept [24], where in the case of travel and tourism participation, people are motivated to continue travel after SCI based on personal (intrinsic) and environmental (external) factors. Shi et al. [25] indicated that travelers with disabilities have their own unique motivations, and concluded they consider travel a way to regain independence, which would help satisfy the psychological need for autonomy. Important motivational forces in travel, leisure, and sport participation for PwSCI include demonstrating skill (where the psychological need for competence could be fulfilled) and bringing them in contact with others [26,27] and better connecting with their family members [28] (where the psychological need for relatedness could be satisfied). Tourism for PwSCI is also regarded as a complex interaction among factors of body function, activity participation, and the environment, where some assume that this population may need to sacrifice travel regardless of their sense of competence or motivation [29]. Recognizing that people often enjoy and benefit from traveling to other places even when facing challenges, SDT theorists have also explained how people with disability may avoid activities because they lack motivation when facing related obstacles [26]. Unfortunately, not much research on autonomy as a motivational factor for tourists with disabilities has been conducted. There is a lack of understanding of the participation of people with disability, and such disinterest might trace back to the complex and interactive components that need to be in place for PwSCI to travel, a setting where individual autonomy is typically not well supported by tourism scholars. We, therefore, propose the following hypothesis:

**H1:**  *Psychological needs satisfaction of competence will have a significant positive impact on travel and tourism participation of PwSCI*.

**H2:**  *Psychological needs satisfaction of autonomy will have a significant positive impact on travel and tourism participation of PwSCI*.

**H3:**  *Psychological needs satisfaction of relatedness will have a significant positive impact on travel and tourism participation of PwSCI*.

### 2.3. Life Satisfaction of PwSCI after Travel Participation

Self-determined participation represents an individual’s ability to exert choice and control, which exhibits their level of regained mobility and further creates meaningful improvements in lifestyle. Travel participation is an important source and component of people’s life satisfaction, as it provides a degree of freedom from work and control exerted by routine. Tourism in particular is perceived to be engaging and arousing and requires a certain sense of mastery while allowing for a certain degree of spontaneity [30]. Specifically, travel service experience and consumption experience during travel were found to contribute to travelers’ overall life satisfaction as well [31,32,33,34]. Moreover, tourist activities can positively enhance life satisfaction by providing social opportunities, such as social support and interactions, and fostering the conditions needed to form close relationships and friendships [35,36]. 

Due to travel constraints that PwSCI perceive or experience, however, they may not see or appreciate the value of tourism, where its voluntary nature may lead them to think they can forego travel to avoid the difficulties they encounter in the process. Further, health status is particularly important for people with disabilities, and in terms of life satisfaction [37], travelers often feel healthier after travel activities [37,38]. The long-term effects of travel include helping one stay active and live a healthy lifestyle [35]. That is, travel participation can impact the satisfaction of life perceptions of people with disabilities in more dimensions than people without disabilities. Finally, travel and tourism scholars have largely been concerned with those who participate rather than those who are excluded from participating [39]. While limited, some research on the impact of travel and tourism on the life satisfaction of PwSCI has been conducted in recent years (e.g., [5,6,20,40]). We thus propose the following hypothesis:

**H4:**  *Travel and tourism participation will positively contribute to the life satisfaction of PwSCI*.

## 3. Methodology

**Figure 1:**
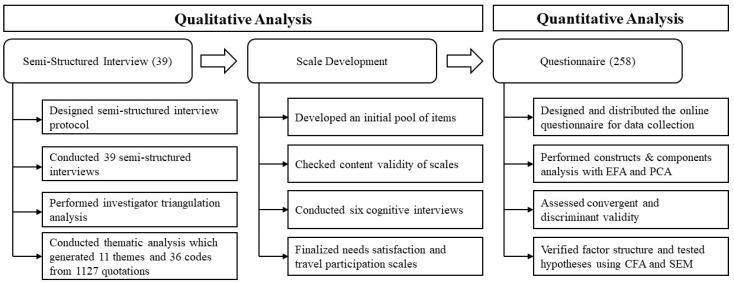
Flow diagram of methodology process.

The study adopts a mixed-method approach, where both qualitative and quantitative data were collected and analyzed separately. Qualitative analysis was conducted to explore the key constructs of psychological needs satisfaction of travelers with SCI and further develop the scale that is specific to PwSCI to make up the missing scale in this area. Quantitative analysis was then conducted to test the developed scale and the hypotheses. The results were compared, combined, and integrated to include generalizable and externally valid insights into the relationships among psychological needs satisfaction for autonomy, competence, relatedness, travel participation, and life satisfaction. The methodology process is shown in Figure 1.

### 3.1. Qualitative Analysis

The qualitative data were collected through semi-structured in-depth, telephone interviews with 39 participants conducted between May and August 2020. Most interviews lasted between 30 and 60 min. Participants were asked to describe their travel experiences and perceptions of experiencing competence, autonomy, and relatedness in the context of travel. Respondents were conveniently recruited from two SCI model systems and communities. The sample size was determined based on the content saturation of the interviews, which were recorded and professionally transcribed. 

The qualitative data collected were thematically analyzed and coded, and investigator triangulation was performed to enhance credibility and dependability and reduce personal biases in analysis and interpretation. First, the responses were coded by two coders at the same time, while a third coder further confirmed or invalidated interpretations. Each quote was manually coded to needs satisfaction- or travel participation-related codes/themes, after which the interpretations were verified by two auditors. In conducting this second round of interpreting all indicator descriptions and distinctions in the analyses, inconsistent findings were also recoded where necessary. ATLAS.ti was adopted for the analysis, a specialized qualitative data management software employed by major brands and academics [41]. The results of the qualitative analysis were further used to develop and validate scales following the rigorous process of scale development, as suggested by DeVellis and Thorpe (2021) [42]. 

### 3.2. Quantitative Analysis

**Figure 2:**
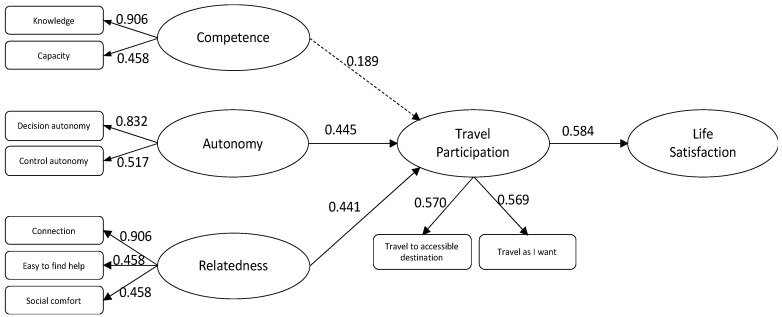
Self-Determination Model of Participation in Travel-Related Activities for PwSCI.

Once the scales were revised and finalized, additional quantitative data were collected from Craig Hospital in Colorado, the Shepherd Center in Atlanta, and SCI community groups to include people who were newly injured as well as those with long-term SCI. The data were analyzed to explore the constructs and provide robust results to prepare for a quantitative examination of the reliability and validity of the scales developed. A structural equation modeling (SEM) using *R* and *Lavaan* was further adopted to test the hypothesized model, which investigates the travel participation of SCI travelers, as illustrated in Figure 2.

## 4. Results

### 4.1. Qualitative Analyses 

Demographic information and injury characteristics of the interviewees are summarized in Table 1. The average age of the interviewees was 53.7 years old, ranging from 22 to 73 years old. Other than one person who had SCI at birth, the rest of the interviewees became injured for various reasons; and the average number of years living with SCI was 24.4 years, ranging from 4 to 50 years. Approximately 70% of the interviewees were male and most were Caucasian (74.4%). Nearly one-third (30.8%) were on disability, 17.9% were employed full time, and 15.4% were retired. The annual household income was polarized: about one-fourth (25.6%) of the interviewees had an annual income of less than $20,000 while 23.1% had more than $100,000. Finally, nearly all participants needed assistive devices in daily living, and most used manual wheelchairs (69.2%), followed by power chairs or scooters (23.1%). 

A total of 11 themes and 36 codes were generated from 1127 quotations. Each quote was manually coded for the three needs satisfaction factors and travel participation. Based on the results of the thematic analysis, several steps were carried out when creating the needs satisfaction and travel participation scales for PwSCI. First, qualitative results derived in the study served as the basis for item writing of the scales to measure the SDT constructs [43] and travel participation. The initial item pool was created to assess each of the four defined constructs. All items were measured with a 5-point Likert scale ranging from 1 = strongly disagree to 5 = strongly agree. Then, the initial item pool was further reviewed and evaluated by experts in accessible tourism for people with disabilities. Finally, cognitive interviews were conducted among six out of the original 39 interviewees to ensure clarity and validity of the items. Minor changes were made to finalize the scales, with 9 items remaining to measure competence, 8 items to autonomy, 11 items to relatedness, and 8 items to travel participation. Life satisfaction was measured with the existing 5-item satisfaction with life scale (SWL) developed by Diener et al. (1985) [44].

Quantitative data were collected next through an online survey using Qualtrics. Data collection was from November 2021 to January 2022, where a total of 258 usable questionnaires were kept for data analysis and hypothesis testing. Descriptive statistics of the constructs in the hypothesized model are displayed in Table 2.

Exploratory factor analysis (EFA) was then conducted to determine the underlying dimensions of the newly developed scales measuring the constructs of three needs satisfaction and travel participation of PwSCI. Principal component analysis (PCA) estimation with varimax rotation was performed using SPSS on items measuring each construct. Two factors were extracted from items measuring the needs satisfaction of competence, explaining 65.39% of the total variance. One item was deleted due to low communality. Four of the remaining eight items appear to measure “travel knowledge” while the other four are related to “travel capacity”. Needs satisfaction of autonomy has two factors: one measures “decision autonomy” and the other refers to “having control”, explaining a total variance of 78.04%. Three factors were extracted from the items measuring relatedness needs satisfaction, explaining a total variance of 58.6%, slightly under the minimum criteria of 60%. Considering the communality values of all items exceeding 0.40, the construct was considered as well-defined by the factor solutions [45]. The first factor appears to be related to “connection with others”, the second factor to “easy to find help”, and the third to “social comfort”. For travel participation, two factors were extracted, accounting for 51.79% of the total variance. Again, considering the communality values of all items exceeding 0.40, the construct was considered as well-defined by the factor solutions. The two extracted factors are related to “travel to accessible destination” and “travel as I want”, respectively.

Composite reliability (CR) and average variance extracted (AVE) were then conducted to examine the scales’ validity. All CR values for multi-item scales were more than the minimum criteria of 0.70. While some variables on the AVE performed poorly, AVE below 0.50 is adequate if CR is above 0.70, statistically indicating a sufficient level of convergent validity [45,46]. Further, a discriminant validity test of the scale was conducted. The square root of AVE for all constructs was above the correlation coefficients of each construct with the other constructs, indicating that the discriminant validity is confirmed [46] (Table 3).

### 4.2. Quantitative Analysis

Demographic and injury characteristics of the survey respondents are summarized in Table 4. Respondents were largely between 31 to 40 years old (22.5%) or over 60 years old (20.2%). About one third of the respondents were injured between 21 to 30 years old (34.1%). There were twice as many male respondents (67.4%) as female respondents (32.6%), a gender skew that is consistent with the national PwSCI data in 2022 [47].

Confirmatory factor analysis (CFA) was conducted to verify the factor structure of the observed variables set, and structural equation modeling (SEM) was conducted to examine the goodness of fit of the measurement model and to test the hypotheses. Table 5 presents model fit information of CFA, where the indices show that the model fit to data is good, which means the data are adequate for SEM analysis. The structural model fits the data adequately as well, as it passes the chi-square test ($\chi^{2}=155.556,\;p<0.05$), with a good RMSEA (0.073) and adequate CFI (0.931) and TLI (0.910), hence confirming the construct validity of the developed scale.

Standardized regression estimates of the tested model are shown in Table 6. The results reveal that both needs satisfaction of relatedness (*β* = 0.445, *p* < 0.05) and autonomy (*β* = 0.441, *p* < 0.001) are significantly related to travel participation of PwSCI, supporting H2 and H3. The relationship between needs satisfaction of competence and travel participation tested as insignificant (*β* = 0.189, *p* > 0.05), hence H1 is not supported. Travel participation was tested as significantly related to life satisfaction (*β* = 0.584, *p* < 0.001), therefore H4 is supported. Figure 2 summarizes the results of all hypotheses tested.

## 5. Discussion

Contrary to many motivational studies of people with disabilities that proposed the development of competences (e.g., affirming/demonstrating skills and knowledge) is fundamental to travel pursuit [51], this study finds that for PwSCI, competence as a need satisfaction did not directly influence their travel and tourism participation behavior. For one thing, the effectiveness of competence in the SDT has been primarily demonstrated among risk-avoidance goal pursuit behaviors (i.e., dieting and tobacco abstinence), whereas it exerts less influence on risk-taking goal pursuits [52], which can include travel and tourism. Additionally, for people with disabilities, travel activities can indeed be risky, where the complex interdependencies between body function challenges, activity participation limitations, and environmental obstacles [29] can reduce the motivation for travel and tourism. For example, interviewees of this study pointed out the many inaccessible designs of accessible facilities they had experienced. The sense of helplessness that many people with disabilities feel when considering traveling may come from the perception that [15] travel failures are inevitable, whether or not they felt competent in traveling. This can lead to reduced motivation to engage in tourism activities, which might explain the inefficient significance of competence in this situation.

The psychological needs satisfaction of both relatedness and autonomy are found to have significant impacts on travel and tourism participation of PwSCI. First, the positive effect of relatedness confirms that reduced loneliness and the opportunity to rebalance their personal and social resources and circumvent the feeling of exclusion from travel are not only benefits [53] but also important driving forces for PwSCI when considering travel and tourism participation. Second, psychological needs satisfaction of autonomy is also proved to be an effective factor when considering travel and tourism activities of PwSCI, from the SDT perspective. Travel and tourism activities can be very gratifying by offering an escape or break from mundane everyday life [54], and therefore offers PwSCI the opportunity to employ autonomous behaviors as volitional or self-endorsed actions that are fully supported by their own willpower. It is also important to point out that SDT argues that one can be autonomous either when acting collectively or individually [55]. Therefore, having autonomy is not necessarily about what PwSCI can do for themselves, but also about what others can do for them, and in ways that they want it done.

Finally, the study testifies to the positive relationship between travel/tourism participation and life satisfaction. Existing evidence has confirmed that the travel experience, especially for fun and enjoyment in tourism, promotes a range of physical and psychological benefits. While all can benefit from travel participation, people with SCI or mobility challenges (including the elderly) particularly benefit from travel and tourism as a mentally and physically healthy pursuit [6]. This is largely because it reduces their perception of isolation or boredom, allowing them to enjoy public resources (i.e., recreational, educational, and cultural) and enhance their social skills and relationships [56,57]. Recent research has also indicated that travel is a key indicator of successful rehabilitation for PwSCI [58], as it may further improve their sense of competence and autonomy in solving issues and maintaining coping strategies when managing obstacles in daily life.

## 6. Conclusions

Results of this study show that increased participation of PwSCI in tourist activities can create meaningful improvements in their daily lives and overall life satisfaction. In looking at the psychological factors that facilitate travel motivation, participation, and outcome, and how these antecedents and consequences interact, findings highlight the need and approaches to empower PwSCI so that they can enjoy the benefits of travel and tourism as much as others. The results of this study further confirm that the psychological needs satisfaction of autonomy and relatedness significantly contribute to self-determined participation in travel and tourism activities of PwSCI. First, while researchers have often treated the benefits of engaging in tourism as travel motivations of people with limited mobility, this study points out that PwSCI do not experience such benefits unless their psychological needs are met. In the same direction, despite efforts to promote barrier removal to ensure travel pursuits of PwSCI, researchers and tourism professionals should realize that intrapersonal inhibitors may have more impact on their travel participation than environmental obstacles. Second, SDT has been successfully applied to a wide variety of life domains, yet the effectiveness of perceived competence in motivating travel participation shown in this study is not found to be significant for travelers with SCI. That is, travel barriers related to psychological needs fulfillment are important challenges that must be removed or successfully managed to accommodate this population.

Theoretical Implications. By applying SDT to the setting of travelers with SCI and emphasizing the importance of psychological factors and processes, we propose a “framework” for the self-determination of PwSCI that highlights the importance of both the autonomy and relatedness perception of PwSCI. The study finds that SDT may not be able to fully explain the psychological process of participation of people with disabilities, as findings of the study show that not all needs satisfaction leads to positive outcomes. For example, competence was not found to contribute to the travel participation of PwSCI. This could be due to the fact that some individuals with SCI depend on their caregivers’ or family/friends’ support to participate in travel and tourism activities. Thus, their own competence needs may not be the most salient in the context of travel and tourism. Further, there is no implicit antagonism between achieving autonomy and receiving support from travel companions, even though it can be very frustrating to balance all the components that need to come together for PwSCI when traveling. Therefore, researchers should reconsider the applicability of SDT in areas where people must cope with complexities related to physical limitations. Theories that better serve the actual needs of people with disabilities, and thus benefit from the expanded theoretical perspectives of this study, must be further explored and established to bring improved life satisfaction and societal involvement of this group.

Practical Implications. The study confirms that creating an autonomy-supportive environment is crucial when servicing customers with physical limitations. It is also important to point out here that people with disability are not a homogeneous group. but a heterogeneous cohort [59] that faces common barriers as well as individualistic impairment needs and concerns. Hence, rather than being committed to making facilities fully accessible to all people, adequate training of service staff when designing services for PwSCI is necessary to supplement the insufficient accessibility of their environment. Second, to improve the accessibility level, we suggest practitioners consult and collect information from customers with disabilities or healthcare professionals, and thus meet the practical needs of customers in real life. Further to this end, this study mainly focuses on PwSCI, yet the results also apply to the elderly and other travelers with limited mobility.

Future research. This study zooms in on the need and approaches of self-determination efforts that can empower PwSCI to gain benefits from tourism participation. This path warrants further exploration on how to fully engage PwSCI in travel opportunities. First, the results confirm that for PwSCI in a travel context, having autonomy is not necessarily about what they can do for themselves, but also about managing how others can assist them in a way that meets their specific needs. Further investigation under this topic on autonomy orientation, autonomy supports, and supportive contexts is therefore recommended. Supportive contexts and environments can be crucial for the successful travel experience of PwSCI, as they provide them with choices and encouragements for personal initiative and support their perceived autonomy in a climate of relatedness. On the other hand, the autonomy orientation and autonomy support of PwSCI could be an interesting topic independent of how supportive the context is, where differences in causality orientations can lead people to have their basic needs met in different ways [60]. Concretely speaking, the quality of interaction with significant others, such as family members, caregivers, or service providers, can affect the degree to which PwSCI feels autonomous, competent, and related to other people, and further affects their overall experience and pro-travel behaviors.

Second, disability in people can significantly compromise their chance to socially integrate, and travel activity has great potential to provide social opportunities. Research is needed on the topic of the mechanisms of building social networks through travel, how to access different types of social supports from each network member, different impacts by the sort of communication (positive/negative/combative), network density as well as fragmentation, etc. In addition, it goes without saying that the support needs of PwSCI are quite different in both intensity and frequency, and in being influenced by the extent of congruence between capacity and the context. Hence, future research on how PwSCI can manage support needs to develop self-determined motivation when utilizing SDT would be meaningful. This outcome is evident when these individuals perceive that they have been provided with opportunities for choice and options, respect in managing their choices, and acknowledgment of their opinions and feelings when trying to meet special needs during travel.

## Figures and Tables

**Table 1:** Demographic and Injury Characteristics of Qualitative Research Sample.

Person	Gender ^1^	Age	Years Since Injury	Work Status	Race	Annual Household Income	Assistive Device ^2^
PSCI01	M	53	35	NA	NA	$40,000–$59,999	WC
PSCI02	M	64	24	NA	Asian, Pacific Islander	<$20,000	WC
PSCI03	M	50	50	NA	White, Caucasian	$80,000–$99,999	WC
PSCI04	F	73	5	NA	White, Caucasian	>$100,000	C/W
PSCI05	F	59	35	Retired	Native American	<$20,000	WC
PSCI06	M	57	40	Part time employed	White, Caucasian	$40,000–$59,999	PC/S
PSCI07	F	56	36	NA	White, Caucasian	>$100,000	WC
PSCI08	M	55	5	NA	Multiracial	>$100,000	WC
PSCI09	M	66	29	NA	Multiracial	>$100,000	WC
PSCI10	M	68	40	NA	White, Caucasian	>$100,000	WC
PSCI11	M	67	40	Retired	White, Caucasian	$40,000–$59,999	WC
PSCI12	F	41	15	On disability	Black, African American	<$20,000	WC
PSCI13	F	56	19	Full time employed	White, Caucasian	>$100,000	WC
PSCI14	F	70	70	Retired	White, Caucasian	$20,000–$39,999	PC/S
PSCI15	F	37	17.5	Full time employed	White, Caucasian	$20,000–$39,999	PC/S
PSCI16	M	50	33	On disability	White, Caucasian	<$20,000	WC
PSCI17	F	43	26	Self-employed	White, Caucasian	$20,000–$39,999	WC
PSCI18	M	67	15	On disability	Hispanic	<$20,000	PC/S
PSCI19	M	54	32	On disability	White, Caucasian	<$20,000	PC/S
PSCI20	F	58	15	Self-employed	White, Caucasian	$20,000–$39,999	WC
PSCI21	M	24	5	Full time employed	White, Caucasian	$40,000–$59,999	WC
PSCI22	M	63	4	Full time employed	White, Caucasian	>$100,000	NA
PSCI23	M	30	2	On disability	White, Caucasian	<$20,000	WC
PSCI24	M	24	3.3	Part time employed	White, Caucasian	<$20,000	WC
PSCI25	F	61	45	Retired	White, Caucasian	$40,000–$59,999	WC
PSCI26	M	48	20	On disability	White, Caucasian	$20,000–$39,999	NA
PSCI27	F	54	26	On disability	White, Caucasian	$20,000–$39,999	WC
PSCI28	M	52	30	On disability	Black, African American	<$20,000	PC/S
PSCI29	M	58	41	Full time employed	White, Caucasian	>$100,000	WC
PSCI30	M	49	10	On disability	Hispanic	<$20,000	WC/PC/S
PSCI31	M	64	46	On disability	White, Caucasian	$20,000–$39,999	WC
PSCI32	M	22	4	Part time employed	White, Caucasian	$60,000–$79,999	WC
PSCI33	M	60	11	On disability/Retired	Multiracial	$40,000–$59,999	PC/S
PSCI34	M	59	30	Part time employed	White, Caucasian	$80,000–$99,999	PC/S
PSCI35	M	65	12	On disability	White, Caucasian	$80,000–$99,999	WC
PSCI36	M	68	42	Retired	White, Caucasian	>$100,000	WC
PSCI37	M	72	36	Retired	White, Caucasian	$40,000–$59,999	PC/S
PSCI38	F	25	22	Full time employed	White, Caucasian	$20,000–$39,999	WC
PSCI39	M	51	25	Full time employed	White, Caucasian	$60,000–$79,999	WC

Note: ^1^ F = female, M = male; ^2^ WC = wheelchair, PC/S = power chair or scooter, C/W = cane or walker; NA = unknown/would rather not say/declined.

**Table 2:** Descriptive Statistics and Covariance Matrix of Variables.

Measured Variables and Measurements		Covariance					Mean	Std. Deviation	CR	AVE	Cronbach’s α
Competence									0.879	0.479	0.721
Travel knowledge	When it comes to travel, I know what works and what does not work for me.	0.638					4.28	0.799	0.752	0.479	
	I am aware of things/situations I do not have control over during travel.	0.21	0.755				4.09	0.869			
	I know the right questions to ask for travel service personnel to meet my needs.	0.233	0.239	0.684			4.03	0.827			
	I make sure I voice my concerns when travel services fail to meet my needs.	0.092	0.216	0.134	0.821		4.06	0.906			
Travel capacity	(Reversed) I sometimes do not know what to do when travel services fail to meet my needs	1.356					3.16	1.160	0.813	0.522	
	I am quite experienced at traveling long distances.	0.309	1.299				3.72	1.140			
	I am good at problem solving during travel.	0.424	0.286	0.577			4.15	0.760			
	I know where to find helpful information for my trips.	0.425	0.439	0.298	0.913		3.75	0.960			
Autonomy									0.903	0.540	0.776
Decision autonomy	I feel free to decide for myself when and to where I want to travel.	1.245					3.88	1.116	0.854	0.544	
	I feel I am in complete control while traveling regardless of whether the service/place is accessible to me.	0.428	1.216				2.90	1.103			
	I feel free to choose what to do when I travel.	0.665	0.631	1.123			3.65	1.060			
	I generally feel I am in control of my own travel.	0.508	0.43	0.388	0.865		3.86	0.930			
	I feel I can pretty much be myself when traveling.	0.429	0.386	0.45	0.404	0.998	3.85	0.999			
Having Control	(Reversed) I need to rely on others to make travel plans for me.	1.309					3.83	1.14	0.774	0.533	
	Doing research and planning ahead of the trip makes me feel in control of my own travel.	0.143	0.402				4.36	0.634			
	I feel free to express my own ideas when making decisions about my travel.	0.217	0.181	0.403			4.36	0.635			
Relatedness									0.923	0.524	0.765
Connection	I feel comfortable asking for help from strangers during travel.	1.198					3.55	1.094	0.787	0.516	
	I am happy to meet new people during travel.	0.256	0.592				4.21	0.769			
	I feel connected to people with whom I travel.	0.196	0.262	0.704			4.01	0.839			
	I feel respected by people I meet on a trip.	0.35	0.294	0.326	0.753		3.72	0.868			
	I feel I can easily connect with the people I meet during travel.	0.362	0.291	0.29	0.434	0.655	3.85	0.809			
Easy to find help	There is someone around to help me travel to places.	1.231					3.82	1.110	0.789	0.557	
	I have someone to discuss my travel plans with if needed.	0.506	0.844				4.11	0.920			
	(Reversed) It is difficult for me to find the help I need to travel long distances.	0.317	0.29	1.231			3.48	1.110			
Social comfort	(Reversed) People I meet during travel often do not engage with me.	1.007					3.65	1.004	0.753	0.505	
	(Reversed) I feel disappointed when people I meet on a trip treat me poorly.	0.33	1.307				2.45	1.143			
	(Reversed) During travel, I often feel people are talking around me but not to me.	0.41	0.401	1.141			3.39	1.068			
Travel Participation									0.887	0.499	0.710
Travel to accessible destination	(Reversed) I try to limit the number of trips I take due to environmental or service barriers for travelers with disabilities.	1.831					3.38	1.353	0.793	0.435	
	(Reversed) I only travel to places that I know are accessible to me.	0.775	1.683				2.59	1.297			
	(Reversed) I avoid traveling to places where I have never been before.	0.442	0.272	1.041			3.95	1.020			
	(Reversed) I mostly travel to places where I do not have to stay overnight.	0.476	0.445	0.386	1.399		3.69	1.182			
	(Reversed) I avoid flying in airplanes when I travel to places	0.64	0.375	0.238	0.41	1.649	3.19	1.284			
Travel as I want	I have been traveling as often as I can.	1.649					3.19	1.284	0.818	0.606	
	My level of participation in travel so far is how I want it.	0.248	1.606				3.13	1.267			
	I feel I have been traveling the way I want.	0.362	0.81	1.596			3.21	1.263			
Life Satisfaction									0.917	0.691	0.833
Life satisfaction	In most ways my life is close to my ideal.	3.735					3.95	1.933			
	The conditions of my life are excellent.	2.622	3.167				4.13	1.780			
	I am satisfied with my life.	2.62	2.328	3.263			4.64	1.806			
	Thus far, I have gotten the important things I want in life.	1.9	1.844	2.069	3.113		4.81	1.764			
	If I could live my life over, I would change almost nothing.	2.087	1.767	2.004	1.764	3.989	3.37	1.997			

**Table 3:** Discriminant Validity Test Results of First Order Constructs.

	Com1	Com2	Auto1	Auto2	Rela1	Rela2	Rela3	TP1	TP2	LS
Com1	0.723									
Com2	0.411 **	0.660								
Auto1	0.503 **	0.276 **	0.738							
Auto2	0.400 **	0.389 **	0.431 **	0.730						
Rela1	0.444 **	0.247 **	0.406 **	0.306 **	0.719					
Rela2	0.242 **	0.191 *	0.235 **	0.197 *	0.359 **	0.746				
Rela3	0.237 **	0.186 *	0.257 **	0.144 *	0.351 **	0.162	0.711			
TP1	0.474 **	0.213 **	0.387 **	0.255 **	0.343 **	0.269 **	0.253 **	0.660		
TP2	0.328 **	0.148 *	0.487 **	0.140 *	0.264 **	0.316 **	0.170 **	0.373 **	0.778	
LS	0.413 **	0.128 *	0.370 **	0.162 *	0.420 **	0.278 **	0.174 **	0.202 **	0.370 **	0.832

Note: Com1 = Travel knowledge, Com2 = Travel capacity, Auto1 = Decision autonomy, Auto2 = Control autonomy, Rela1 = Connection, Rela2 = Easy to find help, Rela3 = Social comfort, TP1 = Travel to accessible destination, TP2 = Travel as I want, LS = Life satisfaction, * *p* ≤ 0.05, ** *p* ≤ 0.01.

**Table 4:** Demographic and Injury Characteristics of Quantitative Research Sample.

Variables	Category	Frequency (Percent, %)	Variables	Category	Frequency (Percent, %)
Age (y)	21–30 y	16 (6.2%)	Family Household Income	Less than $25,000	38 (14.7%)
	31–40 y	90 (22.5%)		$25,000–$49,999	49 (19%)
	41–50 y	42 (16.3%)		$50,000–$74,999	47 (18.2%)
	51–60 y	41 (15.8%)		$75,000–$99,999	45 (17.4%)
	>60 y	52 (20.2%)		$100,000–$124,999	21 (8.1%)
	Declined, Unknown	49 (19.0%)		$125,000 and above	34 (13.2%)
Age at Injury (y)	11–20 y	66 (25.6%)		Declined, Unknown	24 (9.3%)
	21–30 y	88 (34.1%)	Marital Status	Never married (Single)	73 (28.3%)
	31–40 y	38 (14.7%)		Married	125 (48.4%)
	41–50 y	24 (9.3%)		Divorced	26 (10.1%)
	>50 y	37 (14.3%)		Separated	4 (1.6%)
	Declined, Unknown	5 (1.9%)		Widow	5 (1.9%)
Gender	Men	174 (67.4%)		Living with Significant Other/Partner	23 (8.9%)
	Women	84 (32.6%)			
	Other, Unknown	0 (0.00%)		Declined, Unknown	2 (0.8%)
Race/Ethnicity	White, Caucasian	215 (83.3%)	Level of Education	High school or GED or less	35 (13.6%)
	Black, African American	17 (6.6%)		Associate degree	44 (17.1%)
	American Indian, Alaska Native	1 (0.4%)		Bachelor’s degree	69 (26.7%)
	Asian, Pacific Islander	10 (3.9%)		Graduate degree or above	55 (21.3%)
	Other Race, Multiracial	14 (5.4%)		Declined, Unknown	55 (21.3%)
	Declined, Unknown	1 (0.4%)	Total	258 (100%)	

**Table 5:** Summary of Model Fit Indices for the Proposed Model.

	Χ^2^	df	*p*	RMSEA	GFI	CFI	TLI
Suggested value *				<0.08	>0.95	>0.95	>0.95
CFA Model	150.548	67.0	0.000	0.073	0.914	0.932	0.908
Hypothesis Model	155.556	70.0	0.000	0.072	0.912	0.931	0.910

Notes: * Suggested values are based on [48,49,50].

**Table 6:** Summary of the Proposed Model.

Pathways	*β*	Std. Err	z-value	*p* (>|z|)	Test Results
Competence → TP	0.189	0.103	1.258	0.208	H1: Rejected
Relatedness → TP	0.445	0.124	2.526	0.012 *	H2: Accepted
Autonomy → TP	0.441	0.151	2.749	0.006 **	H3: Accepted
TP → LS	0.584	0.336	6.405	0.000 ***	H4: Accepted

Notes: TP = Travel Participation, LS = Life Satisfaction. *** *p* < 0.001, ** *p* < 0.01, * *p* < 0.05.

## Data Availability

The data are not publicly available because data collection is ongoing.
